# An Efficient Synthesis of 3,4-Dihydropyrimidin-2(1*H*)-Ones and Thiones Catalyzed by a Novel Brønsted Acidic Ionic Liquid under Solvent-Free Conditions

**DOI:** 10.3390/molecules20033811

**Published:** 2015-02-26

**Authors:** Yonghong Zhang, Bin Wang, Xiaomei Zhang, Jianbin Huang, Chenjiang Liu

**Affiliations:** 1 Key Laboratory of Petroleum and Gas Fine Chemicals of Ministry of Education, School of Chemistry and Chemical Engineering, Xinjiang University, Urumqi 830046, China; E-Mails: zhzhzyh@126.com (Y.Z.); jbhuang@pku.edu.cn (J.H.); 2 Chengdu Institute of Organic Chemistry, Chinese Academy of Sciences, Chengdu 610041, China; E-Mail: xmzhang@cioc.ac.cn; 3 Physics and Chemistry Detecting Center, Xinjiang University, Urumqi 830046, China; E-Mail: wangbin.yang@163.com

**Keywords:** [Btto][*p*-TSA], Biginelli reaction, dihydropyrimidinones, solvent-free

## Abstract

We report here an efficient and green method for Biginelli condensation reaction of aldehydes, β-ketoesters and urea or thiourea catalyzed by Brønsted acidic ionic liquid [Btto][*p*-TSA] under solvent-free conditions. Compared to the classical Biginelli reaction conditions, the present method has the advantages of giving good yields, short reaction times, near room temperature conditions and the avoidance of the use of organic solvents and metal catalyst.

## 1. Introduction

Over the years, dihydropyrimidinones (DHPMs) and their derivatives have displayed a captivating assortment in natural, synthetic, pharmacological, therapeutic and bioorganic chemistry mainly due to their wide range of biological activities [1,2,3], and they are being studied because of their activities as calcium channel blockers, antihypertensive agents, alpha-la-antagonists and neuropeptide Y(NPY) antagonists [4,5]. Moreover, dihydropyrimidinethiones have been suggested to be useful building blocks for synthesis of natural products, such as the batzelladine family of polycyclic marine alkaloids [6,7], of which batzelladine alkaloids have been found to be potent HIV gp-120-CD4 inhibitors [8,9].

The most simple and straightforward procedure for the synthesis of DHPMs was first reported by the Italian chemist Pietro Biginelli in 1893, it involves a three-component one-pot condensation of benzaldehyde, ethyl acetoacetate and urea under strongly acidic conditions [10]. However, this reaction usually requires harsh conditions, long reaction times and affords low yields, particularly when substituted aromatic and aliphatic aldehydes are employed, which impede their applications. To overcome those disadvantages, several protocols for the synthesis of DHPMs have been developed to improve and modify this reaction by means of microwave irradiation [11,12], ultrasound irradiation [13], ionic liquids [14,15,16], and different types of acidic, base, metal oxide, nanoparticle, enzyme, phase transfer catalysts such as lanthanide triflate [17], H_3_BO_3_ [18], VCl_3_ [19], Sr(OTf)_2_ [20], PPh_3_ [21], Indium(III) halides [22], nanomagnetic-supported sulfonic acid [23], Iron(III) tosylate [24], Bis[(L)prolinato-N,O]Zn-water [25], 1-glycyl-3-methyl imidazolium chloride copper(II) Complex [26], KAl(SO_4_)_2_·12H_2_O supported on silica [27], FeCl_3_-supported nanopore silica [28], SiO_2_-CuCl_2_ [29], metal oxide-MWCNTs [30,31,32], Fe_3_O_3_ and boehmite nanoparticle [33,34], nanosilica-supported tin(II) chloride [35], graphite [36], trypsin [37], silica sulfuric acid [38], Mn(OAc)_3_·2H_2_O [39], Y(NO_3_)_3_·6H_2_O [40], In(OTf)_3_ [41], TaBr_5_ [42], Ce(NO_3_)_3_·6H_2_O [43], silica chloride [44], HCOOH [45], ytterbium chloride [46], TBAB [47] and so on. However, in spite of their potential utility, many of these reported one-pot protocols suffer from drawbacks such as the use of expensive reagents, volatile strong acidic conditions and long reaction times. Therefore, to avoid these limitations, the introduction of a milder and more efficient method accompanied with higher yields is needed.

**Scheme 1:**
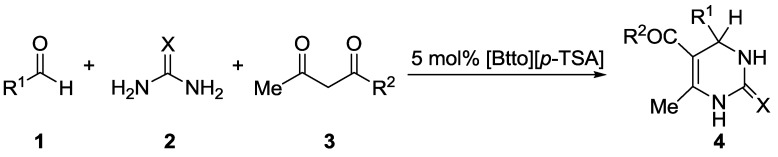
[Btto][*p*-TSA] catalyzed Biginelli reaction.

Lately, Neto and coworker investigated and discussed the crucial role of catalyst and solvent effects for the Biginelli reaction [48]. Furthermore, in recent years, ionic liquids (ILs) are receiving a widespread attention as environmentally acceptable reaction medium or catalyst owing to their advantageous properties [49,50]. In particular, the synthesis of task-specific ILs with special functions according to the requirement of a specific reaction has become an attractive field [51,52]. In previous work, we reported Lewis acid catalyzed Biginelli reaction [53,54,55,56]. Aiming to developing green chemistry, in this work, we wish to report a simple, facile and efficient methodology for the synthesis of 3,4-dihydropyrimidin-2(1*H*)-(thio)ones via condensation of aldehyde, ethyl acetoacetate and urea or thiourea and employing the novel Brønsted acidic ionic liquid [Btto][*p*-TSA] (See Experimental Section) as catalyst for the first time (Scheme 1).

## 2. Results and Discussion

To evaluate the effect of the catalyst under different conditions systematically, we carried out the reaction of benzaldehyde, ethyl acetoacetate and urea as a model reaction and the results are presented in Table 1. Initially the effect of amount of [Btto][*p*-TSA] on the reaction was examined (entries 1–7) and 5 mol % of [Btto][*p*-TSA] afforded the best result (entry 5). Then, the influence of the reaction time on the yield was tested (entries 5, 8–10). It was found that higher yield was obtained when the reaction time was 30 min (Table 1, entry 10). The influence of the reaction temperature on the yield was investigated subsequently (entries 10, 11–12). It was found that 90 °C was still the best reaction temperature (Table 1, entry 10). For the sake of much milder condition to be developed, the reaction temperature at 30 °C was examined at last (Table 1, entries 13–15). The [Btto][*p*-TSA] was also successfully utilized in the Biginelli reaction under near room temperature condition. Although reaction time of 15 h (Table 1, entry 15) gave a little better yield than 10 h, we chose 10 h to save energy. Consequently, the best conditions at 30 °C were a 0.05:1:1:1.5 mole ratio of [Btto][p-TSA], benzaldehyde, ethyl acetoacetate and urea or thiourea for 10 h under solvent-free conditions.

**Table 1:** Effect of catalyst [Btto][*p*-TSA] under different reaction conditions for condensation of benzaldehyde, ethyl acetoacetate and urea ^a^.

Entry	Cat. (mol %)	Temp. (°C)	Time	Yield (%) ^b^
1	1	90	40 min	69
2	2	90	40 min	82
3	3	90	40 min	86
4	4	90	40 min	80
5	5	90	40 min	94
6	6	90	40 min	81
7	10	90	40 min	91
8	5	90	10 min	87
9	5	90	20 min	94
10	5	90	30 min	96
11	5	80	30 min	89
12	5	100	30 min	95
13	5	30	5 h	63
14	5	30	10 h	81
15	5	30	15 h	82

^a^ Reaction conditions: Benzaldehyde (3 mmoL), ethyl acetoacetate (3 mmoL), urea (4.5 mmoL) and catalyst under solvent-free conditions; ^b^ Isolated yield.

In order to study the scope of the procedure, a series of DHPMs were synthesized with the optimized conditions. The results are listed in Table 2. In all cases studied, the three-component reaction proceeded smoothly to give the corresponding DHPMs in excellent yields. Most importantly, aromatic aldehydes carrying either electron donating or electron withdrawing substituents reacted very well to give the corresponding DHPMs with high purity in good yields. Notably, this procedure is compatible with a wide range of functional groups such as methoxy, halides, nitro, hydroxy, *etc*. Beside those above, some sensitive groups also showed to be well tolerated by this method. For instance, furfural and cinnamaldehyde could afford the corresponding products in excellent yields as well. Thiourea has been used with similar success to provide corresponding *S*-dihydropyrimidinones analogues, which are also of interest due to their biological activities (entries 4q–s). The use of methyl acetoacetate as 1,3-dicarbonyl substrate in place of ethyl acetoacetate also gave good to excellent yields, as shown in Table 2 (entries 4t–u).

**Table 2:** [Btto][*p*-TSA]-catalyzed one-pot synthesis of 3,4-dihydropyrimidin-2(1*H*)-ones/thiones at 90 °C ^a^.

Entry	R_1_	R_2_	X	Yields ^b^ (%)	Mp (°C) ^c^	
					Found	Found
4a	C_6_H_5_	EtO	O	96	202–204	206 [26]
4b	4-CH_3_O-C_6_H_4_	EtO	O	97	203–205	205–207 [26]
4c	C_6_H_5_-CH=CH	EtO	O	98	228–230	230–232 [26]
4d	4-F-C_6_H_4_	EtO	O	92	180–183	182–184 [26]
4e	3-Br-C_6_H_4_	EtO	O	99	183–185	185–186 [41]
4f	4-(CH_3_)_2_N-C_6_H_4_	EtO	O	95	250–253	253–254 [19]
4g	3-Cl-C_6_H_4_	EtO	O	97	194–196	193–194 [12]
4h	4-Cl-C_6_H_4_	EtO	O	93	207–210	209–212 [26]
4i	3-O_2_N-C_6_H_4_	EtO	O	92	225–227	227–228 [27]
4j	3-CH_3_O-4-HO-C_6_H_3_	EtO	O	97	232–233	232–233 [38]
4k	2- HO-C_6_H_4_	EtO	O	88	200–202	199–201 [12]
4l	3- HO-C_6_H_4_	EtO	O	98	165–167	167–170 [26]
4m	4-HO-C_6_H_4_	EtO	O	98	224–227	227–228 [27]
4n	3,4-(CH_3_O)_2_-C_6_H_3_	EtO	O	96	173–175	174–176 [27]
4o	2,4-(Cl)_2_-C_6_H_3_	EtO	O	94	250–252	251–252 [12]
4p	2-Furyl	EtO	O	79	205–206	202–204 [12]
4q	C_6_H_5_	EtO	S	98	205–206	207–208 [26]
4r	4-HO-C_6_H_4_	EtO	S	96	200–202	202–203 [26]
4s	4- (CH_3_)_2_N-C_6_H_4_	EtO	S	89	207–209	209–210 [26]
4t	C_6_H_5_	MeO	O	89	211–213	212–213 [26]
4u	3-O_2_N-C_6_H_4_	MeO	O	99	272–275	273–275 [26]

^a^ Reaction conditions: Aldehyde (3 mmoL), β-ketoester (3 mmoL), urea or thiourea (4.5 mmoL), [Btto][*p*-TSA] (0.15 mmoL), with solvent-free conditions, stirred at 90 °C for 30 min; ^b^ Isolated yield; ^c^ Melting points are uncorrected.

At the same time, in order to further investigate this MCR at room temperature, a series of DHPMs were synthesized by using the new reaction set-up at room temperature. The results are summarized in Table 3. It can be observed that all the aldehydes have reacted with β-ketoester and urea at room temperature smoothly to afford the corresponding DHPMs in good to excellent yields. These much milder reaction conditions might make this useful method be promising in biorthogonal chemistry.

**Table 3:** [Btto][*p*-TSA]-Catalyzed one-pot synthesis of 3,4-dihydropyrimidin-2(1*H*)-ones/thiones at room temperature ^a^.

Entry	R_1_	R_2_	X	Yields ^b^ (%)	Mp (°C) ^c^	
					Found	Reported (Lit.)
4a	C_6_H_5_	EtO	O	77	202–205	206 [26]
4b	4-CH_3_O-C_6_H_4_	EtO	O	89	206–208	205–207 [26]
4c	C_6_H_5_-CH=CH	EtO	O	88	230–232	230–232 [26]
4d	4-F-C_6_H_4_	EtO	O	90	180–182	182–184 [26]
4e	3-Br-C_6_H_4_	EtO	O	85	182–185	185–186 [41]
4g	3-Cl-C_6_H_4_	EtO	O	82	190–192	193–194 [12]
4h	4-Cl-C_6_H_4_	EtO	O	85	210–212	209–212 [26]
4i	3-O_2_N-C_6_H_4_	EtO	O	93	225–226	227–228 [27]
4j	3-CH_3_O-4-HO-C_6_H_3_	EtO	O	76	230–233	232–233 [38]

^a^ Reaction conditions: Aldehyde (3 mmoL), β-ketoester (3 mmoL), urea or thiourea (4.5 mmoL), [Btto][*p*-TSA] (0.15 mmoL), with solvent-free condition, stirred at 30 °C for 10 h; ^b^ Isolated yield; ^c^ Melting points are uncorrected.

## 3. Experimental Section

All new compounds were characterized by ^1^H NMR, ^13^C NMR, MS spectra. The ^1^H NMR and ^13^C NMR spectra were obtained on a Varian Inova-400 spectrometer using CDCl_3_, D_2_O as solvent (shown in details in data part) and TMS as an internal standard. LC-MS analyses have been performed on a HP-1100 LC-MS. Melting points were determined using a Büchi B-540 instrument. All melting points are uncorrected.

### 3.1. General Procedure for the Synthesis of 3,4-Dihydropyrimidine-2(1H)-Ones and Thiones

To a mixture of aldehyde (3 mmoL), ethyl acetoacetate (3 mmoL), urea or thiourea (4.5 mmoL) and [Btto][*p*-TSA] (0.15 mmoL) was heated at 90 °C with no solvent for 30 min or at 30 °C under solvent-free condition for 10 h with magnetic stirring. The completion of the reaction was monitored by TLC. After cooling, the reaction mixture was poured onto crushed ice and stirred for 5 min. The separated solid was filtered under suction, washed with cold water thoroughly and then recrystallized from ethanol to afford the pure product. All products are known compounds, which were characterized by mp, IR and ^1^H-NMR spectra. The results are summarized in Table 2 and Table 3.

### 3.2. General Procedure for the Synthesis of [Btto][p-TSA]

1-Butyl-1,3-thiazolidine-2-thione (Btto): Under magnetic stirring, amounts of 1,3-thiazolidine-2-thione (50 mmoL) and tetrabutylammonium bromide (10 mmoL) were dissolved in 30% aqueous solution of sodium hydroxide (22.5 mL), 1-bromobutane (50 mmoL) was added. Then the mixture was heated at 90 °C for 12 h until two phases formed. The upper organic phase and lower water phase were separated with separating funnel. Later, the organic phase was washed with deionized water. Finally, the remaining deionized water was removed under vacuum at 100 °C for 3 h, giving Btto as red liquid.

**Scheme 2:**
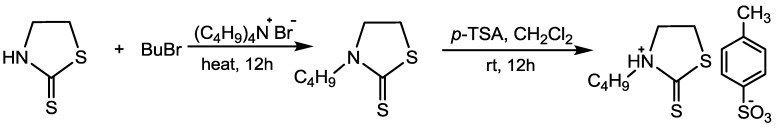
Synthesis of [Btto][*p*-TSA].

1-Butyl-1,3-thiazolidine-2-thione paratoluenesulfate [Btto][*p*-TSA]: Under magnetic stirring, 1-Butyl-1,3-thiazolidine-2-thione (0.02 mol) and paratoluenesulfonic acid (0.02 mol) were mixed with dichloromethane (2 mL) and heated for 12 h at 90 °C. Then, a light yellow liquid zwitterion was formed and washed repeatedly with ether. After dried in vacuum (100 °C, 0.01 Torr), the IL [Btto][*p*-TSA] was obtained (Scheme 2).

### 3.3. Physical and Spectroscopic Data of Products

Compound [Btto][*p*-TSA]: Light yellow liquid. ^1^H NMR (400 MHz, CDCl_3_, TMS): δ: 0.90 (t, *J* = 7.2 Hz, 3H, CH_3_), 1.32–1.45 (m, 2H, CH_2_), 1.65–1.72 (m, 2H, CH_2_), 2.36 (s, 3H, CH_3_), 3.33 (t, *J* = 7.2 Hz, 2H, CH_2_), 3.73 (t, *J* = 8.8 Hz, 2H, CH_2_), 4.50 (t, *J* = 8.4 Hz, 2H, CH_2_), 7.18 (t, *J* = 8.0 Hz, 2H, ArH), 7.76 (t, *J* = 8.4 Hz, 2H, ArH), 7.98 (m, 1H); ^13^C NMR (100 MHz, CDCl_3_, TMS): δ: 13.45, 21.32, 21.45, 30.06, 33.05, 35.51, 55.19, 126.09, 128.88, 140.40, 140.91, 192.28; IR (KBr, *ν*/cm^−1^): 3441, 2960, 2932, 2872, 1741, 1648, 1205, 1183, 870, 687, 568; ESI-MS: *m*/*z* = 176 [M^+^], *m*/*z* = 171 [M^−^].

## 4. Conclusions

In conclusion, we have developed a novel Brønsted acidic ionic liquid [Btto][*p*-TSA] as an efficient catalyst for the synthesis of 3,4-dihydropyrimidin-2-(1*H*)-ones and thiones analogs by multicomponent Biginelli reaction for the first time. Our method offers several advantages such as mild reaction conditions, short reaction times, being environment-friendly and affording good yields.
